# Stimulation of prolactin receptor induces STAT-5 phosphorylation and cellular invasion in glioblastoma multiforme

**DOI:** 10.18632/oncotarget.12840

**Published:** 2016-10-24

**Authors:** Amira Alkharusi, Shengze Yu, Natalia Landázuri, Fahad Zadjali, Belghis Davodi, Thomas Nyström, Torbjörn Gräslund, Afsar Rahbar, Gunnar Norstedt

**Affiliations:** ^1^ Department of Clinical Science and Education, Södersjukhuset, Karolinska Institutet, Stockholm, Sweden; ^2^ Sultan Qaboos University, College of Medicine and Health Sciences, Muscat, Oman; ^3^ School of Biotechnology, KTH - Royal Institute of Technology, Stockholm, Sweden; ^4^ Department of Medicine, Exp Cardiovascular Research Unit and Department of Neurology, Center for Molecular Medicine, Karolinska Institutet, Stockholm, Sweden; ^5^ Department of Women's and Children's Health, Karolinska Institutet, Stockholm, Sweden

**Keywords:** prolactin, prolactin receptor, prolactin receptor antagonist, STAT5, GBM

## Abstract

Glioblastoma multiforme (GBM) is the most common and aggressive primary brain tumor in humans and is characterized with poor outcome. In this study, we investigated components of prolactin (Prl) system in cell models of GBM and in histological tissue sections obtained from GBM patients. Expression of Prolactin receptor (PrlR) was detected at high levels in U251-MG, at low levels in U87-MG and barely detectable in U373 cell lines and in 66% of brain tumor tissues from 32 GBM patients by immunohistochemical technique. In addition, stimulation of U251-MG and U87-MG cells but not U373 with Prl resulted in increased STAT5 phosphorylation and only in U251-MG cells with increased cellular invasion. Furthermore, STAT5 phosphorylation and cellular invasion induced in Prl stimulated cells were significantly reduced by using a Prl receptor antagonist that consists of Prl with four amino acid replacements. We conclude that Prl receptor is expressed at different levels in the majority of GBM tumors and that blocking of PrlR in U251-MG cells significantly reduce cellular invasion.

## INTRODUCTION

Glioblastoma multiforme (GBM) is the most common and aggressive primary brain tumor in humans with a median survival of 15 months despite advanced anti-cancer therapies and surgical intervention [1]. One feature that characterizes GBM is a high level of neovascularization, a product of an imbalance between pro- and anti- angiogenic factors [2, 3]. A number of different alterations of cell signaling components have been found in GBM ranging from receptor signaling to the loss of tumor suppressors [4–7].

Relatively few studies on GBM have concerned the involvement of prolactin (Prl). Prl is a well characterized pituitary hormone and recent studies showed that Prl is also produced outside of the hypophysis in primates, but the role of extra-pituitary Prl production is virtually unknown [8, 9]. In GBM cells, it is interesting to note that anti-angiogenic factors induce local production of Prl as a pro-survival response. A combined exposure of the angiogenic inhibitors endostatin and tumastatin up-regulates Prl receptors (PrlR) in GBM cells through direct action of integrin-targeting factors on tumor cells. This is also the case when human GBM cells are implanted into animals [10]. Furthermore, Prl has been reported to induce a dose-dependent increase in proliferation and survival of G28, G55 and U87-MG human glioma cell lines [10, 11].

One aspect of growth hormone (GH)/Prl actions is related to tissue sensitivity. It is highly relevant to note that certain gene products, e.g. Suppressor of Cytokine Signaling 2 (SOCS2) and Tuberous Sclerosis Complex 2 (TSC2), regulate GH and Prl receptor levels and thereby tissue sensitivity [12, 13]. Both SOCS2 and TSC2 are intracellular proteins that regulate the JAK-STAT and the mTOR pathways respectively. Previous studies have reported an increased STAT and mTOR activity in GBM [14–18]. Studies on SOCS/TSC expression in brain tumors have shown that SOCS1 and SOCS3 are apparently expressed in GBM and play a significant role in the tumor pathogenesis [19]. Of note, loss of TSC1 accelerates malignant glioma genesis when it is combined with oncogenic signals [20]. Patients with tuberous sclerosis have a bi-allelic loss of TSC1/TSC2 and a few clinical cases have been reported on the occurrence of GBM in such patients [21], but further investigations related to this association are needed.

Studies on Prl have shown that it is possible to change particular amino acids to create variants that can still interact with the PrlR but can prevent dimerization and activation of the receptor, thereby blocking PrlR signaling [22]. In this study, we aimed to analyze to which extent Prl regulates GBM cell proliferation and invasion and if a high affinity PrlR antagonist (PrlRA) can affect functions of GBM cells. We also examined expression of the PrlR in human GBM tissues by immuno-histochemistry (IHC).

## RESULTS

### PrlR is expressed at high levels in U251-MG cells and in GBM tissues

**Figure 1:**
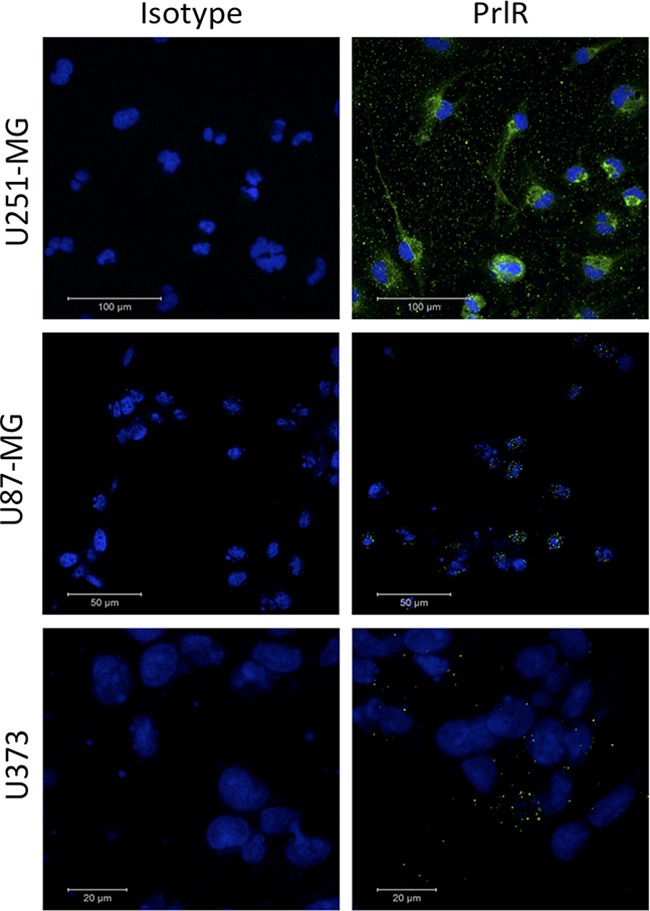
Immunofluorescence visualization of PrlR in U251-MG, U87-MG and U373 cells Cells were grown on coverslips and fixed with methanol. In negative control panel the primary antibody was omitted and the coverslips were incubated with mouse IgG isotype control antibody. Immunofluorescence staining using the PrlR antibody clone 1A2B1. An immunofluorescence signal was observed at intracellular locations. The fluorescence pictures were taken using a confocal microscope.

**Figure 2:**
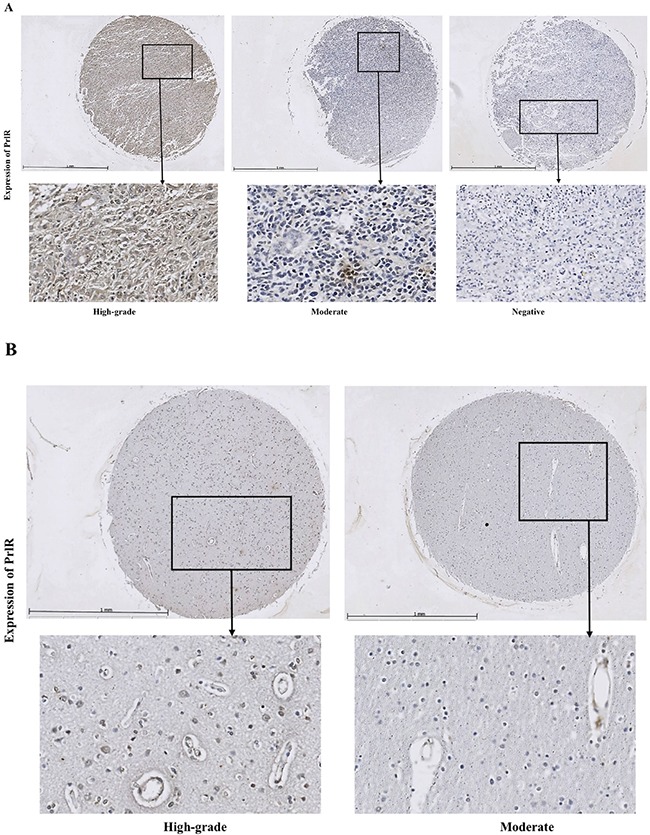

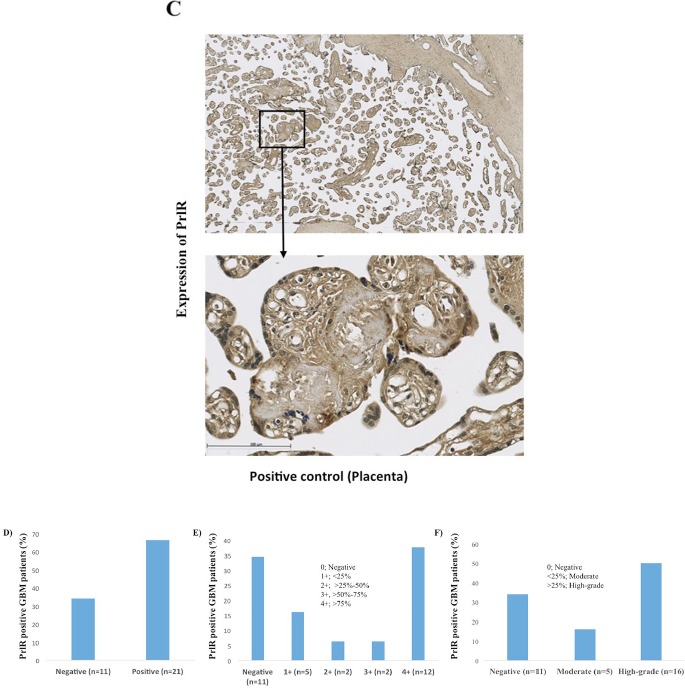
PrlR immuno-reactivity was detected at different levels in GBM tissues Immunohistochemistry was performed for detection of PrlR expression in paraffin embedded tissue micro array (TMA) with 32 cases of GBM tissues and five cases with cancer adjacent normal brain tissues. **A.** Expression of PrlR was detected at high grade as defined by >25% positive cells (left panel), moderate levels as defined <25% positive cells (middle panel) and no expression (right panel). **B.** In cancer adjacent normal brain tissues PrlR expression was detected at high grade (left panel) and moderate levels (right panel). **C.** Detection of PrlR in placenta. **D.** Expression of PrlR could be detected in 66% of GBM patients. **E.** Expression of PrlR was detected at grade 1 (<25%) in 5 out of 32 (16%) patients, at grades 2 (>25-50%) and grade 3 (>50-75%) in 2 out of 32 (6%) and at grade 4 (>75%) in 12 out of 32 (38%) patients. **F.** Expression of PrlR was detected at high-grade (defined as >25% estimated PrlR-expressing cells) in 50% of GBM tumors (16 out of 32), at moderated levels in 16% (5 out of 32) and was not detected in 34% (11 out of 32) of GBM tumors.

PrlR was examined by immunofluorescence (IF) microscopy in cultured U251-MG, U87-MG and U373 glioma cells and IF signals were detected in the cytoplasm of these cells. PrlR was highly expressed in a majority of U251-MG cells, a lower IF signal was detected in a majority of U87-MG cells and in U373 cells the IF signal was almost undetectable (Figure 1). However, PrlR was also expressed at high levels in the peri-nuclear region in some U251-MG cells (Figure 1). The phenomena of peri-nuclear detection of PrlR has been previously seen in other cell types such as rat hepatocytes [23]. A Tissue microarray (TMA) consisting of tissue samples from 32 GBM patients and 5 cancer adjacent normal brain tissues were analysed by IHC to assess the expression of PrlRs (Figure 2A-2B). PrlR was detected at high level in human placenta and used as positive control (Figure 2C). Out of 32 sections, corresponding to 32 GBM cases, 66% were positive for PrlR (Figure 2D). GBM lesions were found to express PrlR at different levels. PrlR was not detected in brain tissues in 11 out of 32 (34%) GBM patients and was detected at grade 1 (<25%) in 5 out of 32 (16%) patients, at grades 2 (>25-50%) and grade 3 (>50-75%) in 2 out of 32 (6%) and at grade 4 (>75%) in 12 out of 32 (38%) patients (Figure 2E). Furthermore, we established a cut off of >25% or <25% estimated PrlR-expressing cells in the brain tissue sections and assigned each section to one of 3 larger groups: negative (no Prl expression), moderate (25%) and high-grade (>25%) expression. Based on this categorization expression of PrlR was not detected in the tissues of 34% (11 out of 32) of GBM patients, but it was detected at high-grade in 50% of GBM tumors (16 out of 32) and at moderated levels only in 16% (5 out of 32) of the tumors (Figure 2F). Expression of PrlR was not detected in cancer adjacent normal brain tissue in one patient and was detected at moderate levels (<25%) in n=2 patients and at high levels (>25%) in 2 another patients.

### Prl induced phosphorylation of STAT5 in U251-MG and U87-MG but not in U373 cells, Induced pSTAT-5 can be blocked with PrlRA

**Figure 3:**
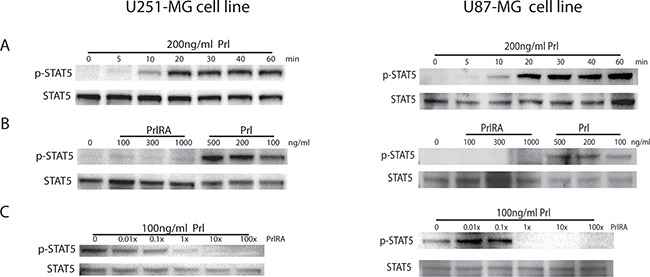
Effect of Prl and a PrlRA on STAT5 phosphorylation in U251-MG and U87-MG cells Cells were kept under serum free conditions over night. **A.** The cells were exposed to 200 ng/ml human Prl or to control PBS for the time period indicated on the X-axis. Following protein extraction and gel electrophoresis, phospho-STAT5 (p-STAT5) and total STAT5 (STAT5) was analyzed using Western blotting. **B.** Serum starved cells were exposed to different concentrations of Prl (100, 200, 500 ng/ml) for 20 minutes. To rule out any agonist activity of the PrlRA, cells were exposed to different concentrations of PrlRA (100, 300, 1000 ng/ml) for 15 min, after which cells were collected for Western blot analysis and probed with antibodies for p-STAT5 and STAT5. **C.** Serum starved cells were exposed to different doses of PrlRA for 5 min, and cells were then exposed to 100ng/ml Prl for 15 min, after which cells were collected for Western blot analysis and probed with antibodies for p-STAT5 and STAT5.

The short-term effect of Prl on intracellular protein phosphorylation was tested in cells at different time points. We detected STAT5 phosphorylation 10 min after exposure to Prl. This phosphorylation reached a maximum of 20 min after exposure to Prl in U251-MG and U87-MG cells but not in U373 cells. Interestingly, the STAT5 phosphorylation remained elevated for the duration of the experiment i.e. 60 min (Figure 3A). In a dose response study, we tested 100-500 ng/ml of Prl and selected 100 ng/ml as a suitable dose to trigger a robust STAT5 phosphorylation (Figure 3B). The PrlRA was tested in this system and, as shown in Figure 3B, the PrlRA alone did not affect STAT5 phosphorylation when added in a dose range of 100-1000 ng/ml. The activity of the PrlRA to block Prl-induced STAT5 phosphorylation was subsequently studied by exposing cells to different concentrations of the PrlRA followed by the addition of 100 ng/ml Prl. As shown in Figure 3C, pre-incubation with 100ng/ml antagonist (equal concentration as Prl) dramatically blocked STAT5 phosphorylation and this was also the case when the concentration of the antagonist was reduced 10-fold.

We could not detect any STAT3 phosphorylation in U251-MG and U87-MG but it was found constitutively active in U373 cells (Data not shown).

### Prl significantly increases invasion only in U251-MG cells and had no effect on proliferation in U251-MG, U87-MG and U373 cells

**Figure 4:**
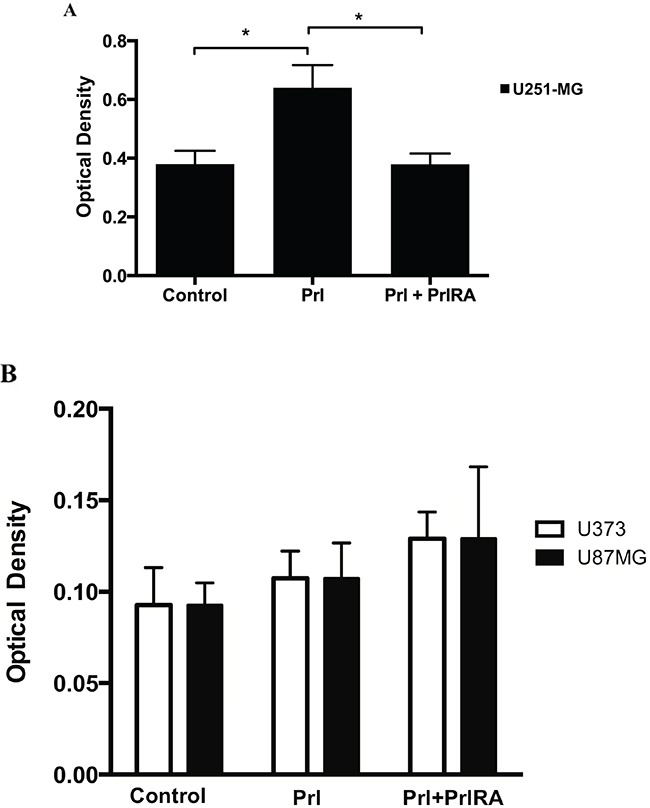
Effect of Prl and PrlRA on cell invasion Cells were cultured under serum free conditions in 24 well cytoselect trans-well plates, then cells were allowed to attach and were cultured with Prl (200 ng/ml), PrlRA (200ng/ml) or with PBS as control. After 48 h, cell extracts were prepared from the layer representing invading cells and optical densities of the extracts were measured at 560 nm. **A.** The PrlRA significantly decreased invasion of U251-MG cells when used in combination with Prl stimulation while **B.** no effect has been detected for PrlRA in U87-MG and U373 cells. All values are mean +/− SEM, * = P-value <0.05.

**Figure 5:**
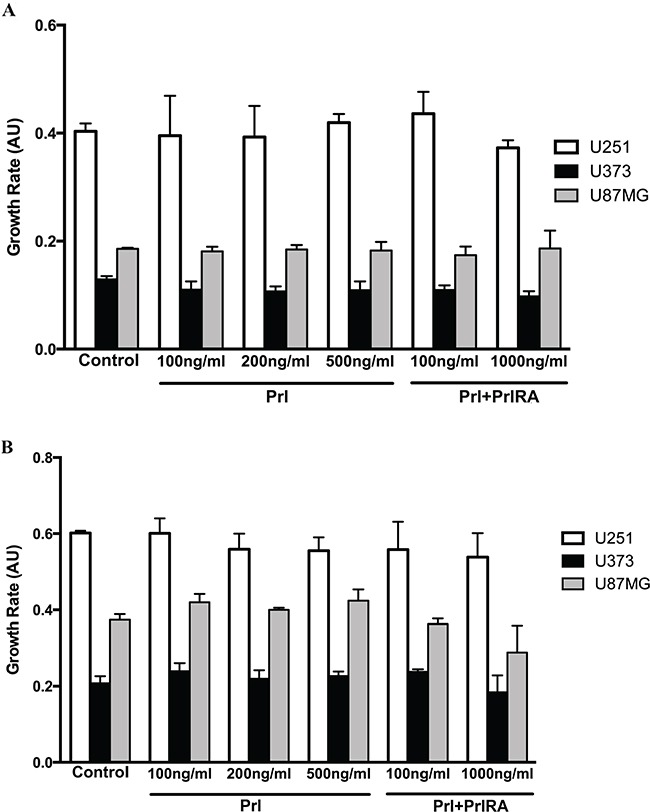
Effect of Prl on U251-MG, U87-MG and U373 cell proliferation Cells were cultured in two different concentrations of serum (1% and 10%). Human Prl was added to sub-confluent cells at a concentration of 200 ng/ml. Cell viability was assessed 3 days later for cells cultured in **A.** 1% or **B.** 10% FBS. The X-axis depicts Prl dose dependent effect on cells growth and the Y-axis shows the relative growth determined by absorbance at 600 nm. Each data point represents triplicate assays. All values are mean +/− SEM.

The invasive properties ofhuman U251-MG, U87-MG and U373 cells were investigated using a Cell Invasion Assay. Compared to untreated cells, we found increased invasion of only U251-MG cells but not U87-MG and U373 cells treated with Prl and this effect could be blocked by the high affinity PrlRA (Figure 4A-4B). Furthermore, proliferation of human U251-MG cells in response to Prl was studied using a colorimetric assay. Under the conditions tested, Prl had no effect on the proliferation of U251-MG, U87-MG and U373 cells (Figure 5).

## DISCUSSION

There is a marked heterogeneity among GBM cell lines both at the cellular and the molecular levels, in this study we have demonstrated high expression of PrlR in majority of U251-MG cells, only low expression in majority of U87-MG cells and at much less extend in U373 cells and at different levels in GBM tissues. Approximately, 66% of GBM patients had PrlR-positive cells in their tumours. In 50% of these patients, PrlR was detected at high-grade (as defined >25% positive cells) in the tumours. Moreover, we found significantly increased levels of phosphorylated STAT5 in Prl treated U251-MG and U87-MG cells but not in U373 cells. Instead in line with previous study a constitutively active pSTAT-3 was detected in all cells [24, 25].

Enhanced activity and invasiveness in response to Prl treatment was found only in U251-MG cells but not in U87-MG and U373 cells. Interestingly, high expression of PrlR was detected in U251-MG cells compared to U87-MG and U373 cells and consequently significant or no invasion capacity of GBM cells in response to Prl may reflect the impact of different levels of PrlR expression on GBM cells. Furthermore, the invasiveness of U251-MG cells could be significantly reduced to that of control cells by using a novel high affinity PrlRA. Furthermore, in a dose response study, Prl showed no effect on U251-MG, U87-MG and U373 cells proliferation. Implicating that highly proliferative GBM cells use other cellular pathways important in proliferation. A number of different alterations of cell signaling molecules have been found to promote angiogenesis/growth in GBM, including increased signaling from Vascular Endothelial Growth Factor receptor (VEGFR), Epidermal Growth Factor receptor (EGFR) [26] and Platelet–derived Growth Factor (PDGF) [2]. In our study, we could show that PrlR, which belongs to the cytokine receptor family, is highly expressed on U251-MG cells compared to U87-MG and U373 cells. Since U251-MG cells respond to Prl, invasion capacity of these cells likely depends on Prl-mediated signalling. It is clear that the U251-MG cells require serum to grow and fetal calf serum, which is commonly used in culturing media of these cells, is likely to contain bovine Prl, even though this hormone is not regarded as a strong ligand of the human PrlR [27]. However, in GBM cells, Prl has been reported to be locally produced [10], which is in congruence with the finding of extra-pituitary Prl production in primates. In primates, but not in rodents, Prl seems to be produced outside of the pituitary gland due to the existence of a separate gene promoter [9, 28]. Extra-pituitary Prl production has elegantly been demonstrated in a mouse model by replacing the mouse Prl promoter with the human version [29]. Data on production of Prl in human brain tissues are scarce. However, one study reported detection of Prl mRNA in human central nervous tissues [30], whereas another study showed Prl expression in GBM detectable with IHC but not with real time PCR [31]. However, mRNA expression in normal human brain tissues for Prl and PrlR genes had been reported in multiple gene databases such as Gene cards. Beside the different possibilities for different sources of Prl, it is relevant to consider the existence of PrlR in brain tissues and GBM. It is interesting to note that the choroid plexus is a rich source of PrlRs [32] that may have a role to transport Prl into the CNS. In terms of GBM, a large-scale transcript profiling experiment detected PrlR mRNA in different glioma cell lines [10, 33, 34]. However, different studies on GBM showed that Prl enhanced intracellular calcium uptake by glioma cells and increased the cellular half-life in GBM cells [35]. Taken together, in the present study, we detected expression of PrlR at different levels in GBM cells and tissues and speculate that expression of PrlR is of relevance for GBM tumors. However, in our study PrlR was also expressed at different levels in cancer-adjacent normal brain tissues, which emphasize a possible indirect effect of the tumor micro-environment on these sites that may affect the expression of PrlR. Finally, it is possible that GBM represents a state of either increased Prl production or increased Prl sensitivity. In support of the latter, loss of TSC2 (a well-known suppressor of mTOR) function leads to increased Prl sensitivity of lymphangioleiomyomatosis (LAM) cells, a sarcoma–like cell type [36, 37]. The mTOR system is an important signal integrator and regulator of protein synthesis and seems to be over-active in GBM [17, 38, 39]. However, mTOR inhibitors have a limited efficacy on human GBM and this could partly be explained by a limited ability of rapamycin to cross the blood brain barrier in humans [3]. Possibly, there is a need of combined targeted treatments, e.g, using both mTOR inhibitors and EGFR inhibitors to treat recurrent GBM. There are studies showing that combining rapamycin, an mTOR inhibitor, with an EGFR inhibitor improves the clinical outcome in a small number of patients with recurrent GBM brain tumor [40–42]. The STAT5 regulation by Prl observed in this study confirms previous studies [43, 44] and activation of this transcription factor seems of particular relevance for glioma cells. STAT5 is a transcription factor that is activated by Prl and phosphorylation of this protein has an anti-apoptotic role in cells. Previous studies have shown that phosphorylated STAT5 mediate oncogenic effects of EGFR [45] in different tumors, including GBM [46, 47]. Theoretically, the use of a combination therapy of angiogenic inhibitors that up-regulate PrlR in GBM with a PrlRA may significantly improve GBM patients' outcomes.

Taken together, we hypothesize that GBM lesions are hypersensitive to endocrine or locally produced Prl and will respond to agents disrupting Prl signals. The availability of the PrlRA presented in this work, which is devoid of any residual agonistic properties, makes studies on this subject feasible.

In conclusion, preventing invasion of GBM cells by blocking of PrlR with a novel high affinity PrlR antagonist may offer a new therapeutic opportunity for treatment of GBM patients in combination with conventional therapy. Future studies are needed to clarify the potential role of PrlRA in GBM treatments.

## MATERIALS AND METHODS

### Cell culture

The cell line U251-MG, U87-MG and U373 were obtained from American Tissue Culture Collection (ATCC, USA). U251-MG and U87-MG Cells were cultivated in DMEM and U373 cells in RPMI. All medium supplemented with 10% Fetal Bovine Serum (FBS) (Gibco, USA), 100 U/ml penicillin and 100 μg/ml streptomycin at 37°C, 5% CO_2_.

Cells were lysed in 50 mM Tris HCl, pH 7.5/ 150 mM NaCl/ 5 mM EDTA/ 0.5% Igepal-40/ 1 mM Na_3_VO_4_/ 20 mM NaF/ 1 mM DTT/ 1 mM PMSF/ 1X Cocktail inhibitor (Complete mini, Roche, Switzerland). Cell debris was removed by centrifugation at 14,000× g for 15 min at 4°C. Cells were treated with recombinant human prolactin (a generous gift from Novo Nordisk A/S Denmark). A PrlRA was used [22, 48, 49] at different concentrations as specified in the figure legends to block the receptor. Control cells were treated with phosphate buffered saline (PBS) solution in all experiments.

### Western blot

Whole cell lysates were separated in SDS/PAGE gels and transferred to polyvinylidenediflouride (PVDF) membranes (Invitrogen, Carlsbad, CA, USA). After blotting membranes were blocked in 5% BSA (Sigma–Aldrich, St. Louis, Missouri, USA) in Tris-Buffered Saline (TBS) containing 0,1% Tween 20. Membranes were incubated with one or more of the following antibodies as specified in the figures and figure legends: antibodies to detect phosphorylated and total STAT5, were obtained from (Cell Signaling, Danvers, MA, USA). Antibody dilution is 1:1000 and membranes incubated with primary antibody at 4°C overnight. When indicated, incubations with the appropriate HRP-conjugated secondary antibody (Cell Signaling) were conducted with dilution of 1:5000 for 1 hour at room temperature. Membranes were visualized with the ECL Western blotting detection system (Millipore) according to the manufacturer's instruction and were analysed by using a DSS camera (Bio-Rad).

### Immunofluorescence staining and confocal laser scanning microscopy

For immunofluorescence staining, the cells were allowed to grow on coverslips and cultured in complete medium at 37°C, 5% CO_2_. Cells were fixed with ice-cold methanol for 10 min; then excess methanol was removed. After three subsequent washes with PBS, the cells were blocked with protein blocker (Dako Cytomation, Denmark) and then incubated with the primary antibody mouse anti-PrlR clone 1A2B1 in dilution of 1:50 (Invitrogen) over night at 4°C, washed 3 times with PBS; and then incubated for 1 h at room temperature in the dark with the secondary antibody Alexa fluor 488- conjugated goat anti-mouse IgG (Invitrogen). Finally, the slides were washed, mounted with Vectra shield containing DAPI (H-1200, Vector Laboratories, CA, USA) and stored at 4°C in the dark. Controls for specificity and background staining were performed by incubating samples with mouse IgG antibody. Confocal Laser Scanning Microscopy (CLSM) (Zeiss, Germany) was used to obtain the images.

### Immunohistochemical analyses of PrlR expression in GBM tissues

To investigate the expression of PrlR in human GBM tissues, we analyzed a paraffin embedded tissue micro array (TMA) with 32 cases of GBM and five cases with cancer adjacent normal brain tissues (Biomax, USA, cat no. BS17016). Paraffin embedded tissue sections from human placenta served as control for staining. Working conditions were optimized for heat-induced antigen retrieval, after deparaffinization and rehydration of tissues as described before [50]. For antigen retrieval, the slides were treated with citrate buffer (pH 6.0, BioSite, Täby, Sweden) for 3 min at 90°C and thereafter for 120 min at 50°C in a water bath. Endogenous biotin was neutralized using the Avidin-Biotin Blocking Kit (Dako, Glostrup, Denmark). Sections were blocked for endogenous peroxidase (3% H_2_O_2_, for 15 min, Sigma, USA) and for Fc receptor with FC receptor blocker (30 min, at 20°C; Innovex Biosciences, CA). Then slides were incubated with a primary antibody specific for PrlR (clone 1A2B1, Invitrogen), diluted in a common antibody diluting buffer (PMC BioGenix, TN, USA) and incubated at 4°C over night followed by repeated washing and incubation with biotinylated anti mouse secondary antibody diluted according to the manufacturer's instructions (BioGenix) for 45 min at room temperature, followed by incubation with streptavidin-biotin-peroxidase complex (BioGenix). Haematoxylin was used for counterstaining. For negative controls, mouse IgG isotype control (Dako) was used and the primary antibody was omitted. Negative controls were performed in parallel to all experiments. Sections were scanned using Hamamatsu Nano Zoomer-XR Digital slide scanner C12000 and visualized using Nano Zoomer Digital Pathology (NDP) viewer software (U12388-01; NDP.view2 Viewing). Grading of estimated number of positive cells within tissue sections was performed as described before [51–53]. The number of cells in the tissues expressing PrlR was estimated and each tissue was scored as: negative; 0, <25%; 1, >25%-50%; 2+, >50%-75%; 3+ and >75%; 4+. Based on the possible impact of percentage number of the cells expressing PrlR in the tissues, we used a cut off > or <25% estimated PrlR expressing cells in the tissues and made 3 larger groups: negative; no cells expressed PrlR, moderate; <25% of the cells expressed PrlR and high-grade; >25% of the cells expressed PrlR.

### Cell proliferation assay

GBM cells were seeded into 96-well culture plates at a density of 1000 cells per 200-uL well and were allowed to attach. Then medium was replaced and cells were grown in low serum (1%) or in high serum (10%) and exposed to Prl or PrlRA (generous gift from Novo Nordisk A/S Denmark). Cell viability was assessed 3 days later for cells cultured in 1% or 10% FBS, respectively, with the Cell Titer 96 An aqueous Non-Radioactive Cell Proliferation Assay (Promega Biotech, Nacka, Sweden) according to the manufacturer's instructions.

### Invasion assay

The GBM cells were starved over night by omitting FBS from the culture medium. The invasive properties of tumor cells were analyzed using CytoSelect™ Cell Invasion Assay kit (Cell Biolabs, San Diego, CA, USA). In this method, 0.5 × 10 ^6^ cells were suspended in serum free culture medium and plated into the invasion wells following kit instructions. Then Prl and PrlRA (200 ng/ml each) were added to the medium as specified in the figure legend. The invasive properties of the cultured cells were analyzed according to the manufacturer's instructions. The OD of the extracts were measured at λ= 560 nm. Growth charts were created on the basis of the obtained absorbance values.

### Prl receptor antagonist

A changed amino acid in human Prl at position 129, where Gly is substituted for Arg, generates a protein (hPrl-G129R) that blocks the Prl receptor [22, 48]. Studies have shown that Prl G129R competes with exogenous Prl when added in >10-fold molar excess to compensate for its lower affinity for the PrlR [48, 49]. In the present study we used a protein with increased affinity to block the receptor, Prl S33A, Q73L, G129R, K190R [54].

### Statistical analysis

Cell culture experiments were performed in duplicates or triplicates in at least three independent experiments. Statistical significance of the differences was evaluated using unpaired, 2-tailed Student's *t*-test, ANOVA test with post-hoc analysis, and was considered significant when the significance level of the test was p < 0.05.
